# ACT001 Suppresses the Malignant Progression of Small‐Cell Lung Cancer by Inhibiting Lactate Production and Promoting Anti‐Tumor Immunity

**DOI:** 10.1111/1759-7714.70028

**Published:** 2025-02-27

**Authors:** Xiao‐Jing Ding, Ting Mei, Xiao‐Nan Xi, Jing‐Ya Wang, Wen‐Jing Wang, Yue Chen, Ya‐Xin Lu, Ting‐Ting Qin, Ding‐Zhi Huang

**Affiliations:** ^1^ College of Pharmacy Nankai University Tianjin China; ^2^ National Clinical Research Center for Cancer Tianjin Medical University Cancer Institute & Hospital Tianjin China; ^3^ Key Laboratory of Cancer Prevention and Therapy Tianjin China; ^4^ Tianjin's Clinical Research Center for Cancer Tianjin China; ^5^ Department of Thoracic Oncology, Tianjin Lung Cancer Center, Tianjin Cancer Institute & Hospital Tianjin Medical University Tianjin China; ^6^ State Key Laboratory of Medicinal Chemical Biology Nankai University Tianjin China; ^7^ College of Chemistry Nankai University Tianjin China

**Keywords:** ACT001, aerobic glycolysis, lactate, PGK1, small‐cell lung cancer, tumor associated macrophages

## Abstract

**Background:**

Improving the “cold” tumor immune microenvironment (TIME) of small‐cell lung cancer (SCLC) represents a promising therapeutic approach. The metabolite lactate plays a crucial role in shaping the immune‐cold tumor microenvironment (TME) and facilitating tumor progression. Phosphoglycerate kinase 1 (PGK1) is a key enzyme involved in tumor lactate metabolism. This study demonstrates that ACT001 improves the TIME of SCLC through inhibiting lactate production by targeting PGK1.

**Methods:**

The cytotoxic effects of ACT001 on SCLC cell lines NCI‐H1688 and NCI‐H446 were evaluated using MTT assay, clone formation, EdU incorporation, wound healing, and invasion assays. To elucidate the mechanism of action of ACT001, proteomic techniques, pull‐down assays, LC–MS/MS, surface plasmon resonance, immunofluorescence, lactate generation, glucose uptake, and western blot assays were conducted. A xenograft model was used to assess the in vivo anti‐tumor activity of ACT001.

**Results:**

ACT001 inhibited the proliferation, invasion, and metastasis of SCLC both in vitro and in vivo. Additionally, it reduced lactate accumulation and M2 macrophage polarization. Mechanistically, ACT001 released micheliolide, which covalently modified Cys316 of PGK1 under physiological conditions. This suppressed PGK1 activity and restored the distribution of PGK1 in mitochondria and the cytoplasm under hypoxic conditions.

**Conclusions:**

ACT001 inhibits the malignant progression of SCLC by suppressing lactate production, modulating macrophage polarization, and restraining tumor metastasis through PGK1 targeting.

## Graphical abstract

ACT001, exerted anti‐tumor activity, inhibited lactate production and promoted anti‐tumor immunity by targeting PGK1, showing no observable systemic toxicity in vivo.

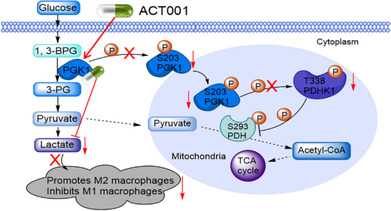

## 1 Introduction

Small‐cell lung cancer (SCLC) is a formidable, relatively immune‐resistant, and highly lethal subtype of lung cancer [1, 2, 3, 4, 5]. Approximately 80% of patients with SCLC initially respond well to the first‐line standard treatment, which combines etoposide with platinum [6]. However, the majority of patients experience relapse or develop resistance within a short period [7, 8]. While immune checkpoint inhibitors (ICIs) have improved survival in 10%–20% of cases [9, 10, 11, 12, 13, 14, 15, 16, 17, 18, 19], their efficacy remains insufficient to justify their widespread use. There is an urgent need to elucidate the mechanisms underlying the malignant progression of SCLC and to develop novel therapeutic strategies.

Malignant tumor cell metabolism exhibits distinct characteristics, particularly an increased conversion of glucose to lactate. Cancer cells predominantly rely on glycolysis for energy production, even in the presence of abundant oxygen, a phenomenon termed aerobic glycolysis [20, 21, 22]. This process results in lactate fermentation within the cytoplasm rather than oxidative phosphorylation in mitochondria. The excessive lactate secreted by cancer cells creates an acidic microenvironment, which promotes tumor cell proliferation and immune evasion [23, 24, 25]. Notably, lactate mediates interactions between various cell populations within tumor tissues, modulating immune surveillance functions, such as the infiltration and M2 polarization of tumor‐associated macrophages [26, 27, 28, 29, 30, 31]. Consequently, targeting these unique metabolic pathways presents a promising strategy for reshaping the tumor microenvironment (TME) and enhancing anti‐tumor immune responses.

Phosphoglycerate kinase 1 (PGK1) is a key enzyme in glycolysis, catalyzing the conversion of 1,3‐diphosphoglycerate (1,3‐BPG) and adenosine diphosphate (ADP) into 3‐phosphoglycerate (3‐PG) and adenosine 5′‐triphosphate (ATP) [32, 33]. PGK1 is overexpressed in various cancer types, promoting aerobic glycolysis, cancer cell proliferation, and metastasis [34, 35, 36]. Post‐translational modifications regulate PGK1 activity. Phosphorylation at Ser203 and O‐GlcNAcylation at Thr255 enhance PGK1 activity and mediate its translocation from the cytoplasm to mitochondria [37, 38]. Upon mitochondrial translocation, PGK1 phosphorylates pyruvate dehydrogenase 1 (PDHK1), reducing pyruvate dehydrogenase activity and limiting pyruvate entry into mitochondria for oxidative phosphorylation [37]. This highlights PGK1's role as a protein kinase in coordinating glycolysis and the tricarboxylic acid cycle, which is critical for cancer metabolism and tumorigenesis. Acetylation at the Lys323 site of PGK1 enhances liver cancer proliferation and tumorigenesis [39]. Furthermore, macrophage‐associated PGK1 phosphorylation promotes aerobic glycolysis, indicating PGK1's role in the immune microenvironment [40].

ACT001, a novel drug developed by Accendatech Co. Ltd. (Tianjin, China), has been designated as an orphan drug for glioblastoma by the FDA. This compound gradually releases micheliolide (MCL) under physiological conditions, thereby enhancing MCL's oral bioavailability. ACT001 demonstrates potent inhibitory and immunomodulatory effects across various tumors by suppressing multiple signaling pathways, including signal transducer and activator of transcription 3 (STAT3), nuclear factor kappa‐B (NF‐kappa B), toll‐like receptor 4 co‐receptor MD2, and plasminogen activator inhibitor‐1 (PAI) [41, 42, 43, 44, 45, 46, 47, 48, 49]. Furthermore, when used for treating brain metastases from SCLC, ACT001 has been included in the breakthrough treatment list by the center for drug evaluation (CDE) in China (Handling Number: CXHL2000167). In this study, we discovered that ACT001 releases MCL, which directly targets and inhibits PGK1, revealing a novel mechanism through which ACT001 modulates tumor metabolism, tumor immune microenvironment (TIME), and cancer progression.

## 2 Methods

### 2.1 Cell Lines

NCI‐H1688 and NCI‐H446 cell lines were obtained from Accendatech Co. Ltd. (Tianjin, China), while the GFP‐H1688 and THP‐1 cell lines were acquired from the American Type Culture Collection (ATCC).

### 2.2 Expression and Purification of PGK1 Wild‐Type and Mutant Proteins

PGK1 and its mutant protein were expressed and purified according to previously established experimental protocols [37].

### 2.3 Surface Plasmon Resonance Assay

The interaction between ACT001 and MCL with PGK1 was assessed using surface plasmon resonance (SPR) following previously established protocols [45].

### 2.4 Proteomic Analysis

The samples were processed according to previously established protocols and were subsequently reconstituted in a 0.1% formic acid aqueous solution for analysis using LC–MS/MS (Thermo Fisher Scientific, MA, USA) [47].

## 3 Results

### 3.1 ACT001 Inhibited the Proliferation, Invasion and Migration of SCLC In Vitro

The cytotoxic effects of ACT001 and MCL on SCLC cell lines NCI‐H1688 and NCI‐H446 were evaluated using MTT assays over 72 h. Both compounds exhibited cytotoxicity, with ACT001 demonstrating IC_50_ values of 19.99 and 28.68 μM, respectively, and MCL showing IC_50_ values of 3.49 and 6.43 μM, respectively (Table 1).

**TABLE 1:** MTT assay results of ACT001 and MCL on NCI‐H1688 and NCI‐H446 cell lines for 72 h.

Drugs/cell types	NCI‐H1688	NCI‐H446
ACT001	19.99 ± 1.75 μM	28.68 ± 2.56 μM
MCL	3.49 ± 0.70 μM	6.43 ± 2.27 μM

**FIGURE 1:**
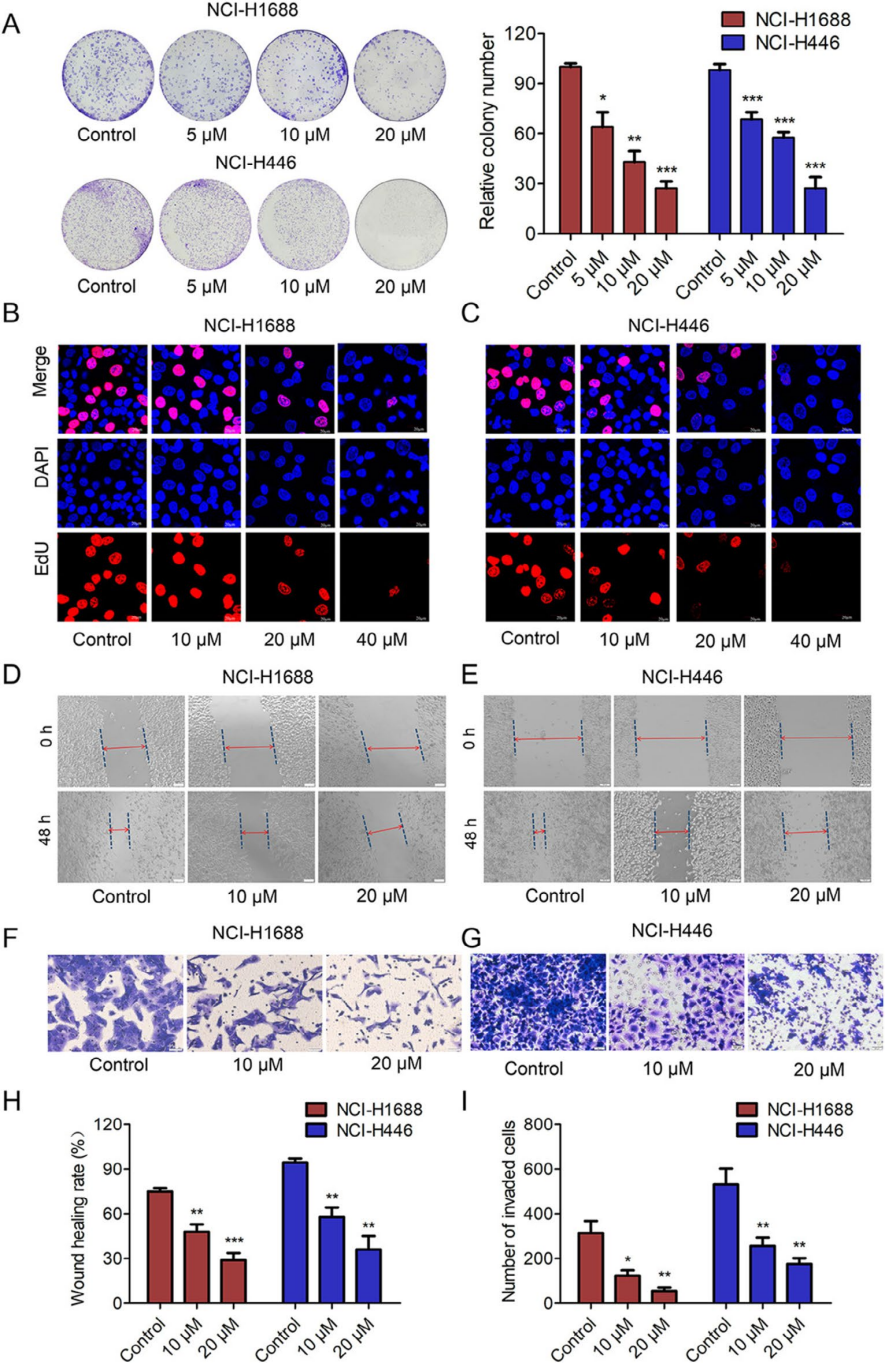
ACT001 inhibited the proliferation, invasion, and migration of SCLC cells. (A) Image depicting ACT001's inhibition of clone formation in NCI‐H1688 and NCI‐H446 cells. (B, C) EdU assay results demonstrating reduced DNA replication in SCLC cells treated with ACT001. (D–G) Concentration‐dependent inhibitory effects of ACT001 on invasion and metastasis of NCI‐H1688 and NCI‐H446 cells. (H, I) Quantitative analysis of invaded cell numbers and wound healing rate (%) for NCI‐H1688 and NCI‐H446 cells. Data are represented as mean value ± SD (*n* = 3, *p < 0.05, **p < 0.01, ***p < 0.001).

We further evaluated the effect of ACT001 on the proliferation of SCLC cells. Clone formation experiments and EdU cell proliferation assays indicated that ACT001 inhibited clone formation (Figure 1A) and DNA replication (Figure 1B,C) in SCLC cells. Additionally, ACT001 significantly reduced the wound healing rate and the number of invaded cells in NCI‐H1688 and NCI‐H446 cell lines in a dose‐dependent manner compared to the control group (Figure 1D–I). These findings confirm that ACT001 exerts various anti‐SCLC effects, including the inhibition of proliferation, metastasis, and invasion in NCI‐H1688 and NCI‐H446 cells.

### 3.2 ACT001 Reduced SCLC Aerobic Glycolysis In Vitro

**FIGURE 2:**
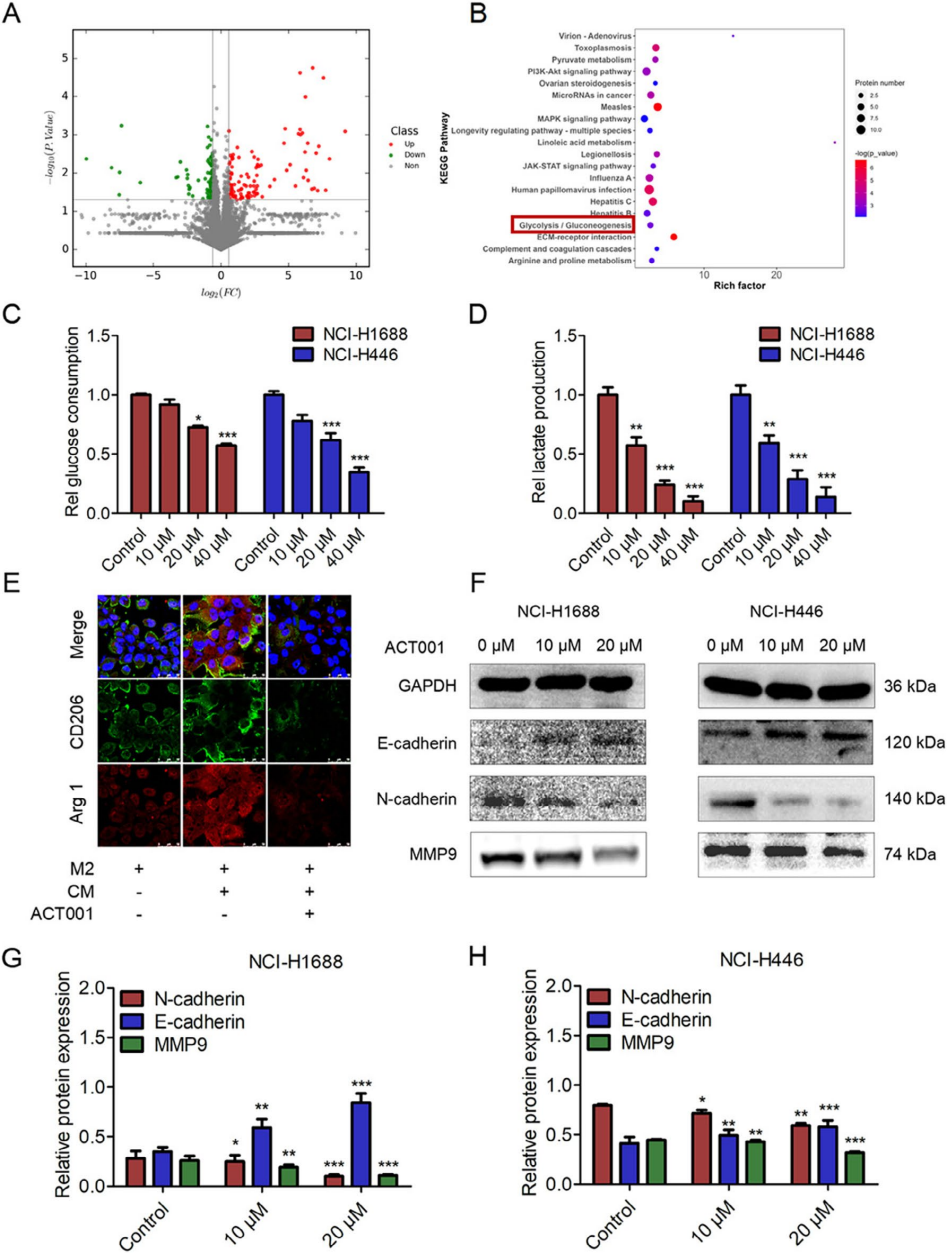
ACT001 regulated aerobic glycolysis in SCLC cells. (A) Differentially expressed proteins between the control and ACT001‐treated groups (24 h). Red dots indicate upregulated proteins and green dots indicate downregulated proteins. (B) KEGG enrichment analysis of differentially expressed proteins. (C, D) ACT001 inhibited glucose uptake and lactate production in NCI‐H1688 and NCI‐H446 cells. (E) Representative images of M2 and CM‐incubated M2 macrophages with and without ACT001 treatment, stained with antibodies against Arg1 (red), CD206 (green), and DAPI (blue). (F) Western blot analysis of E‐cadherin, N‐cadherin, and MMP9 levels in NCI‐H1688 and NCI‐H446 cells treated with ACT001 at the indicated concentrations for 48 h. (G, H) Quantitative analysis of protein expression levels. Error bars represent mean ± SD, *n* = 3. *p < 0.05, **p < 0.01, ***p < 0.001.

To elucidate the mechanism of action of ACT001 against SCLC, proteomic experiments were conducted to determine the proteomic changes in NCI‐H1688 cells with and without ACT001 treatment. A fold change > 1.5 or < 0.06 and *p* < 0.05 were used as the cutoff criteria (Figure 2A). Kyoto Encyclopedia of Genes and Genomes (KEGG) enrichment analysis revealed that the glycolysis/gluconeogenesis pathway was regulated by ACT001 (Figure 2B). Lactate production and glucose uptake experiments were performed to evaluate the regulatory effect of ACT001 on glycolysis in NCI‐H1688 and NCI‐H446 cells. ACT001 treatment decreased glucose consumption and lactate production in both cell lines (Figure 2C,D). Notably, at a concentration of 10 μM, ACT001 significantly inhibited lactate levels without substantially altering glucose uptake in tumor cells, suggesting that ACT001 modulates the metabolic pathway of SCLC cells.

Elevated lactate leads to an acidic TME and promotes the polarization of M2 macrophages [26]. Given ACT001's significant inhibition of lactate production, we further assessed its effect on lactate‐stimulated M2‐type polarization of macrophages. Human THP‐1 monocytes were stimulated with phorbol 12‐myristate 13‐acetate, interleukin‐4, and interleukin‐13 to represent M2 macrophage polarization (Figure 2E). Immunofluorescence assays confirmed that conditioned medium (CM) from NCI‐H1688 cells increased M2 macrophage marker levels; conversely, ACT001 inhibited CM‐facilitated M2‐type polarization (Figure 2E). This experiment demonstrated that ACT001 can suppress the CM‐induced polarization of M2 macrophages. Additionally, the glycolytic metabolite lactate promotes tumor metastasis through epithelial‐to mesenchymal transition (EMT) [50]. We examined the expression of EMT‐related markers in SCLC cells incubated with or without ACT001. Western blot analysis showed that ACT001 significantly inhibited N‐cadherin and MMP9 levels while increasing E‐cadherin expression (Figure 2F–H). These experiments indicate that ACT001 regulates glycolysis, inhibits lactate production, reduces CM‐induced polarization of M2 macrophages, and suppresses tumor EMT progression in SCLC cells.

### 3.3 MCL Targeted PGK1 and Inhibited PGK1 Enzyme Activity

**FIGURE 3:**
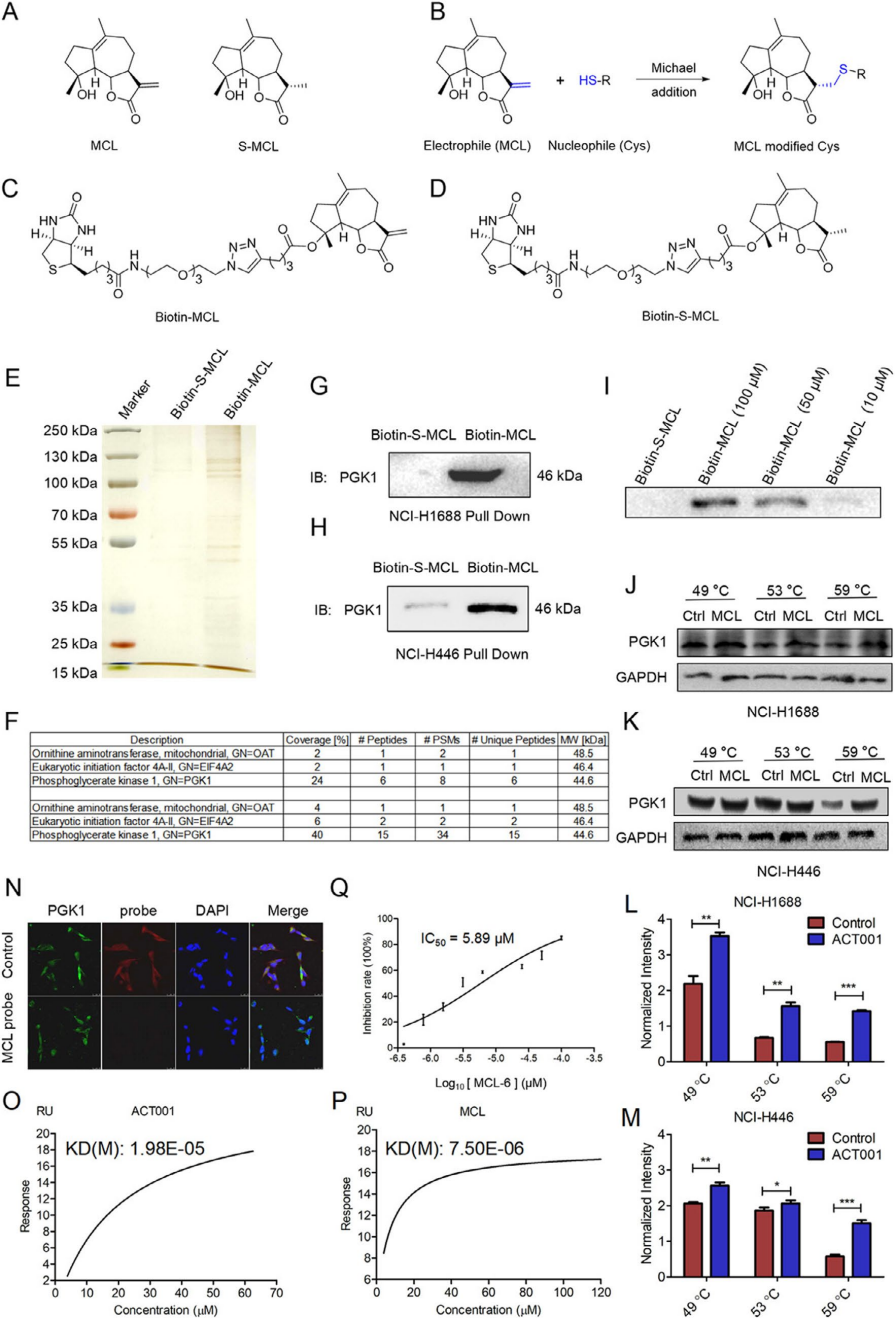
MCL targeted PGK1 and inhibited PGK1 enzyme activity. (A) Structures of active MCL and inactive S‐MCL. (B) Mechanism of MCL binding to cysteine residues. (C, D) Structure of Biotin‐MCL and Biotin‐S‐MCL. (E) Silver staining analysis of target proteins bound to Biotin‐MCL and Biotin‐S‐MCL. The experiments were conducted in duplicate. (F) Initial LC–MS/MS identification of proteins bound to Biotin‐MCL but not Biotin‐S‐MCL (Two independent experiments). (G, H) Western blot analysis demonstrating the Biotin‐MCL probe binding to PGK1 in NCI‐H1688 and NCI‐H446 cells, while the Biotin‐S‐MCL probe did not bind. (I) Concentration‐dependent binding of Biotin‐MCL to PGK1 in NCI‐H1688 cells. (J, K) MCL enhanced the thermal stability of PGK1 protein at elevated temperatures in vitro in NCI‐H1688 and NCI‐H446 cells. (L, M) Normalized intensity analysis of PGK1. (N) Co‐localization of MCL probe (red) and PGK1 (green) in NCI‐H1688 cells. (O, P) SPR results showing the direct interaction between PGK1 protein and ACT001 or MCL. (Q) Inhibitory effect of ACT001 on the wild‐type recombinant PGK1 enzymatic activity. The experiments were performed in triplicate.

Our previous research demonstrated that ACT001 releases the active compound MCL under physiological conditions [48]. MCL functions as a reactive Michael acceptor, forming covalent bonds with cysteine residues of target proteins (Figure 3A,B).

To identify MCL's targets, we employed MCL‐related probes in subsequent experiments. In vitro pull‐down assays were conducted using biotin‐conjugated probes. Proteins bound to the active probe Biotin‐MCL (Figure 3C) or the inactive probe Biotin‐S‐MCL (Figure 3D) in NCI‐H1688 cells were identified through LC–MS analysis (Figure 3E). The analysis revealed that elongation initiation factor EIF4A2, ornithine aminotransferase, and PGK1 were pulled down by Biotin‐MCL but not by Biotin‐S‐MCL (Figure 3F). Among these, PGK1 exhibited the highest coverage and most specific peptide segments (Figure 3F). Based on these findings, we postulated that PGK1 is the primary target of MCL in NCI‐H1688 cells. Western blot and dose‐dependent pull‐down assays confirmed that PGK1 was pulled down by Biotin‐MCL in SCLC cells (Figure 3G–I). A cellular thermal shift assay demonstrated that MCL treatment enhances the thermal stability of PGK1 (Figure 3J–M). Immunofluorescence analysis indicated co‐localization of the MCL probe with PGK1 in NCI‐H1688 cells (Figure 3N). SPR analysis revealed that the dissociation constant (KD) value of ACT001 and PGK1 was 19.8 μM, while the KD of MCL and PGK1 was 7.5 μM (Figure 3O,P). To evaluate whether MCL inhibits PGK1 enzyme activity, wild‐type recombinant PGK1 (rPGK1) protein was expressed and purified. The results indicated that MCL attenuated PGK1 activity with an IC_50_ value of 5.89 μM, suggesting that MCL is a potential novel PGK1 inhibitor (Figure 3Q).

**FIGURE 4:**
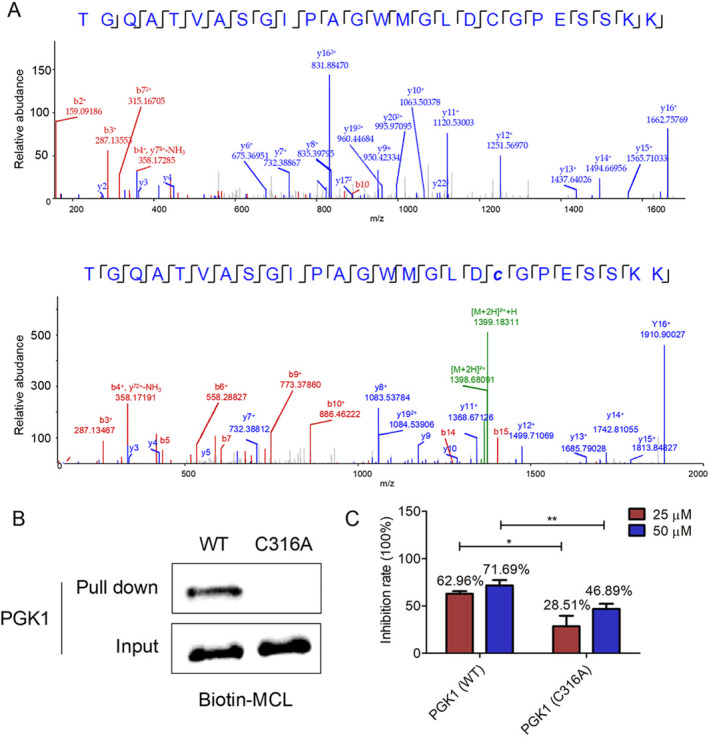
Cysteine 316 was responsible for MCL‐mediated PGK1 inhibition. (A) LC–MS/MS analysis of the Cys316‐containing tryptic peptide in recombinant PGK1 in the absence (top) and presence (bottom) of MCL. (B) Western blot images of recombinant wild‐type PGK1 and its mutants pulled down by Biotin‐MCL. (C) Wild‐type PGK1 and PGK1 C316A proteins were incubated with MCL at the indicated concentrations for 30 min at room temperature for PGK1 activity assay.

Next, we investigated the binding site between MCL and PGK1. rPGK1 was incubated with or without MCL and subjected to LC–MS/MS. Tryptic peptides containing cysteine were evaluated. The mass of the Cys316‐containing peptide TGQATVASGIPAGWMGLDCGPESSKK was measured as 2548.23 Da without MCL and 2796.36 Da with MCL, corresponding to a mass shift of 248.13 Da, which is consistent with the addition of one MCL molecule (Figure 4A). These results suggested that Cys316 residues of PGK1 might be covalently modified by MCL. To confirm this, we mutated Cys316 to alanine (rPGK1 C316A) and performed pull‐down experiments with recombinant cysteine‐mutated PGK1. The results showed that MCL specifically binds to Cys316 of rPGK1 (Figure 4B). Additionally, we investigated whether the Cys316 mutation could impact the inhibitory effect of MCL on PGK1 activity. The results demonstrated that MCL significantly inhibited wild‐type PGK1 activity, but this effect was markedly suppressed in the Cys316‐mutated PGK1 (Figure 4C). Collectively, these experiments demonstrate that MCL directly targets Cys316 of PGK1, forming a covalent bond that inhibits its activity.

### 3.4 ACT001 Reduced Mitochondrial Translocation of PGK1 by Inhibiting PGK1 S203 Phosphorylation

**FIGURE 5:**
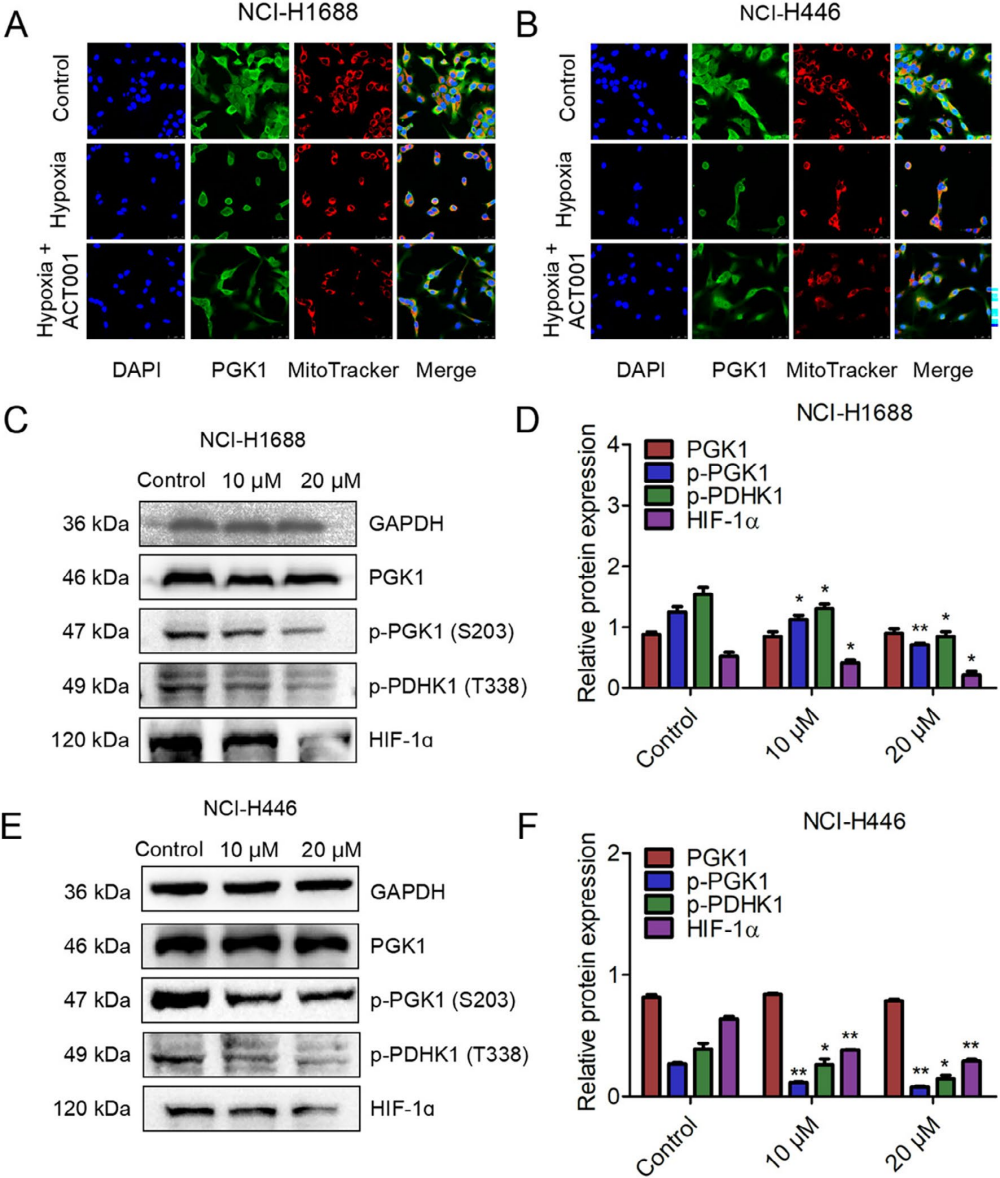
ACT001 reversed PGK1 mitochondrial translocation under hypoxic conditions by inhibiting p‐PGK1 and p‐PDHK1. (A, B) ACT001 treatment reversed PGK1 mitochondrial translocation under hypoxic conditions in NCI‐H1688 and NCI‐H446 cells. (C) Western blot analysis demonstrating the effect of ACT001 at indicated concentrations for 48 h on the expression of p‐PGK1, p‐PDHK1, and HIF‐1α in NCI‐H1688 cells. (D) Statistical analysis of the related proteins in NCI‐H1688 cells. (E) Western blot analysis showing the effect of ACT001 at indicated concentrations for 48 h on the expression of p‐PGK1, p‐PDHK1, and HIF‐1α in NCI‐H446 cells. (F) Quantitative analysis of p‐PGK1, p‐PDHK1, and HIF‐1α expression levels in NCI‐H446 cells. Error bars represent mean ± SD (*n* = 3 biological replicates). *p < 0.05, **p < 0.01, ***p < 0.001.

Cancer cells maintain a high glycolytic rate even in the presence of sufficient oxygen. However, most solid tumors contain hypoxic regions. Hypoxia enhances glycolysis by promoting the conversion of pyruvate to lactate rather than its utilization for mitochondrial oxidation, thereby supporting tumor cell survival and progression [51]. Hypoxia can induce PGK1 translocation to mitochondria, mediated by PGK1 phosphorylation at S203. Mitochondrial PGK1 activates PDHK1 by phosphorylating it at T338. Phosphorylated PDHK1 subsequently inhibits mitochondrial pyruvate metabolism and increases lactate production [37]. To investigate the role of ACT001 in PGK1 mitochondrial translocation, SCLC cells were treated with or without ACT001 to assess PGK1 phosphorylation at S203. Concurrently, immunofluorescence analysis was conducted to visualize PGK1 distribution within cells. Under normoxic conditions, PGK1 was distributed throughout the cytoplasm and nucleus (Figure 5A,B). However, in hypoxic conditions, intracellular PGK1 was completely co‐localized with mitochondria. Notably, treatment with 10 μM ACT001 resulted in PGK1 redistribution to the cytoplasm in NCI‐H1688 and NCI‐H446 cells (Figure 5A,B). These experiments indicated that ACT001 effectively targeted intracellular PGK1 and reduced its mitochondrial translocation under hypoxic conditions. Further western blot analysis revealed that intracellular p‐PGK1(S203) and p‐PDHK1(T388) protein levels decreased with ACT001 incubation in a dose‐dependent manner without altering PGK1 expression (Figure 5C–F). These findings demonstrate that ACT001 reduces PGK1 phosphorylation, inhibits mitochondrial pyruvate metabolism, and indirectly reduces lactate formation.

### 3.5 Anti‐Proliferation, Anti‐Metastatic and Anti‐Glycolysis Effects of ACT001 Were at Least Partially Dependent on the Expression of PGK1

**FIGURE 6:**
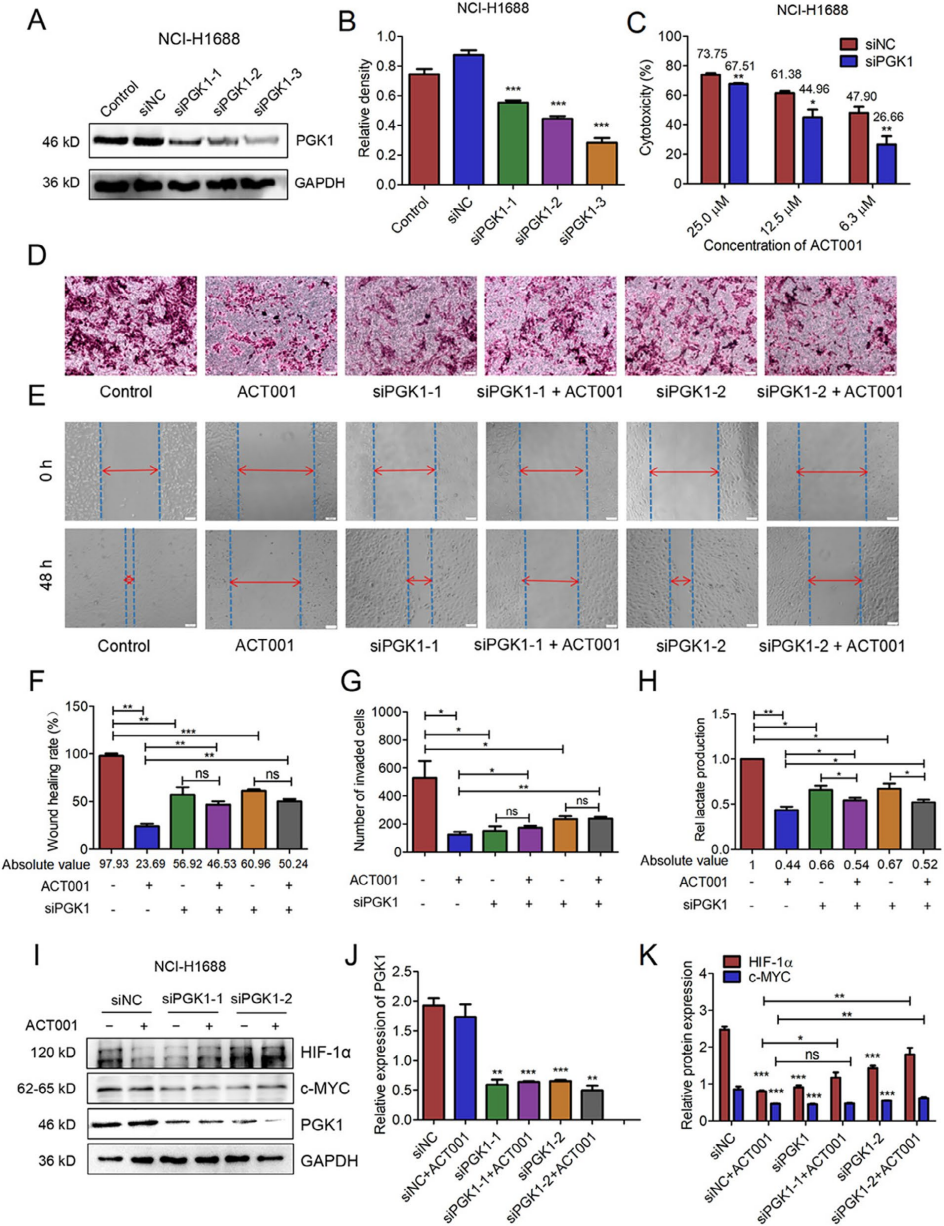
PGK1 knockdown induced resistance to ACT001 treatment in SCLC cells. (A) Western blot results of PGK1 knockdown in NCI‐H1688 cells. (B) Quantitative analysis of PGK1 protein expression. (C) Cytotoxicity assay showing ACT001's inhibitory effect on transfected NCI‐H1688 cells after 72 h. Bar heights represent mean inhibition rates (*n* = 3). (D, E) ACT001 treatment significantly inhibited the migration and invasion capabilities of NCI‐H1688 cells. PGK1 siRNA transfection attenuated this effect. (F, G) Quantitative analysis of wound healing and transwell assay results. (H) ACT001 inhibited lactate production in NCI‐H1688 cells, an effect reversed by PGK1 knockdown. (I) PGK1 depletion or ACT001 treatment inhibited HIF‐1α and c‐MYC expression in NCI‐H1688 cells. ACT001 administration following PGK1 depletion did not further suppress HIF‐1α and c‐MYC expression. (J, K) Quantitative analysis of protein expression levels. Error bars represent mean ± SD (*n* = 3 biological replicates). *p < 0.05, **p < 0.01, ***p < 0.001.

To validate the role of PGK1 on the inhibitory effect of ACT001 on SCLC, we used siRNA to interfere with PGK1 expression in NCI‐H1688 cells. Three different PGK1 siRNAs successfully inhibited PGK1 expression (Figure 6A,B). MTT assays revealed that PGK1‐silenced (siPGK1) cells were more resistant to ACT001 compared to control cells (Figure 6C). In subsequent wound healing and transwell assays, ACT001 inhibited the migratory and invasive capacity of NCI‐H1688 cells, but this inhibitory effect was reduced in the siPGK1 + ACT001 group compared to the ACT001 treatment alone group (Figure 6D–G). Additionally, lactate production was significantly reduced in NCI‐H1688 cells after ACT001 treatment or PGK1 knockdown (Figure 6H). However, the ability of ACT001 to inhibit lactate formation in siPGK1‐NCI‐H1688 cells was diminished (Figure 6H). ACT001 treatment or PGK1 knockdown notably inhibited the expression of HIF‐1α and c‐MYC, two key regulators of glycolysis in SCLC cells. However, compared to the siPGK1 group, ACT001 treatment after PGK1 knockdown did not further reduce the expression of HIF‐1α and c‐MYC (Figure 6I–K). These experiments demonstrate that ACT001 inhibits tumor metastasis and lactate production by targeting PGK1.

### 3.6 ACT001 Delayed the Progression and Diminished Metastasis of SCLC In Vivo

**FIGURE 7:**
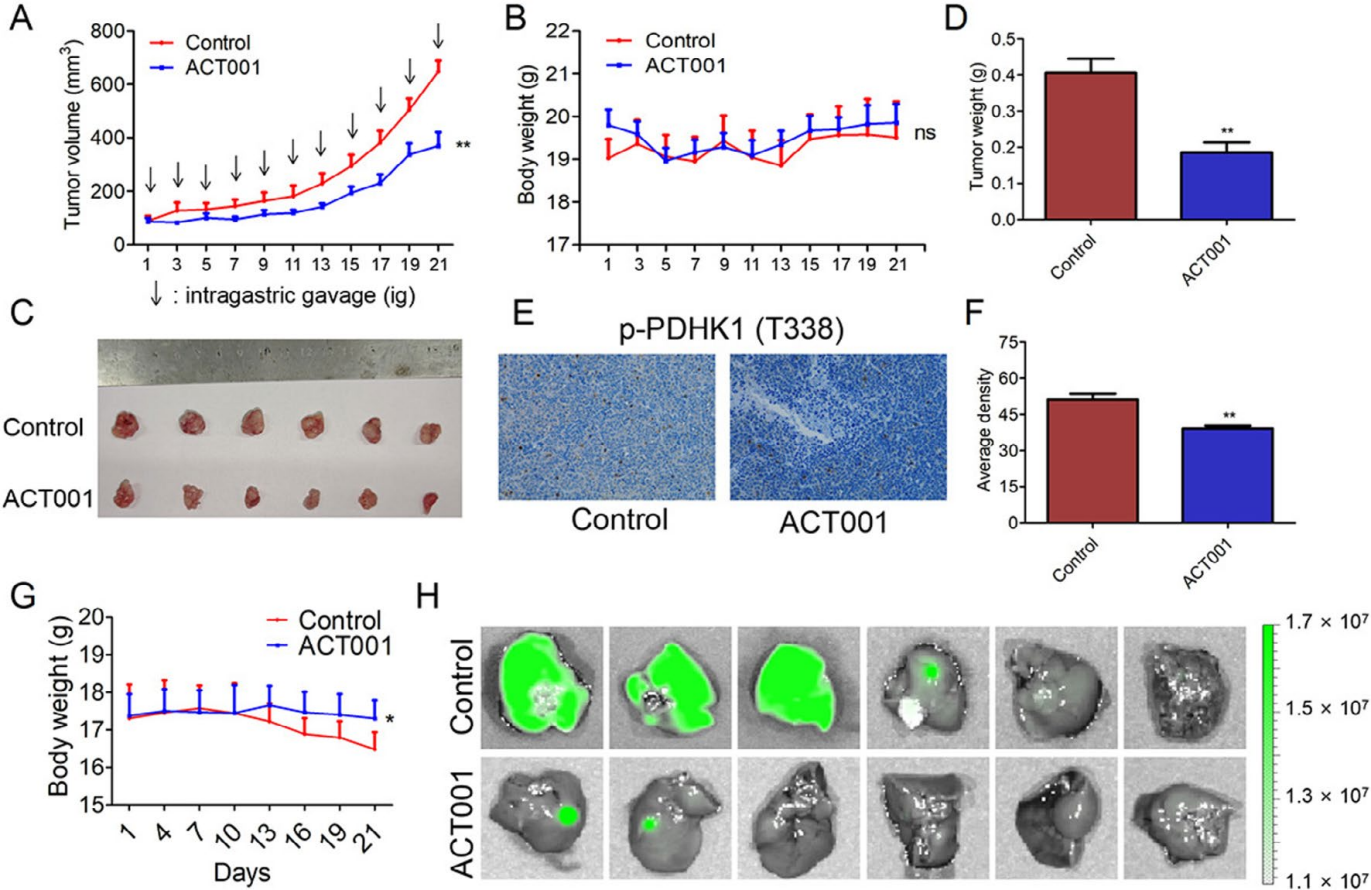
ACT001 suppressed the proliferation and metastasis of small‐cell lung cancer in vivo (ACT001 administered at 200 mg/kg over a 21‐day period, *n* = 6). (A) Tumor growth curve showing the effect of treatment in vivo. (B) Mouse body weight change curve. (C) Tumor collection images from the two groups. (D) Histogram depicting tumor weights. (E, F) IHC images and quantitative analysis of p‐PDHK1 staining in tumor tissues (*n* = 6). (G) Mouse body weight change curve. (H) ACT001 inhibited liver metastasis of GFP‐H1688 cells in Balb/c nude mice.

To evaluate the anti‐tumor effects of ACT001 in vivo, we established a nude mouse tumor‐bearing model using NCI‐H1688 cells. Tumor‐bearing mice were randomly assigned to either the control group or the ACT001 administration group, which received oral administration of ACT001 once daily at a dose of 200 mg/kg. On Day 21, tumor volumes and weights in the ACT001 treatment group were reduced to 56.8% and 45.8%, respectively, compared with the control group (Figure 7A–D), demonstrating that ACT001 effectively reduced disease burden by inhibiting tumor growth. Notably, ACT001 had minimal impact on body weight, following a trend similar to the control group, suggesting no significant side effects (Figure 7B). Furthermore, immunohistochemistry (IHC) results indicated that ACT001 reduced p‐PDHK1 protein expression in vivo (Figure 7E,F). We subsequently assessed the effect of ACT001 on tumor metastasis using tail vein injection of GFP‐H1688 cells. In the ACT001 group, three mice (3/6, 50.0%) exhibited detectable liver metastasis, whereas in the control group, five mice (5/6, 83.3%) displayed evident tumor metastasis (Figure 7G,H). These findings demonstrate that ACT001 treatment can reduce tumor growth and metastasis in vivo.

### 3.7 ACT001 Inhibited Lactate Accumulation, Reduced M2 Macrophage Infiltration, and Enhanced Anti‐Tumor Immune Responses In Vivo

**FIGURE 8:**
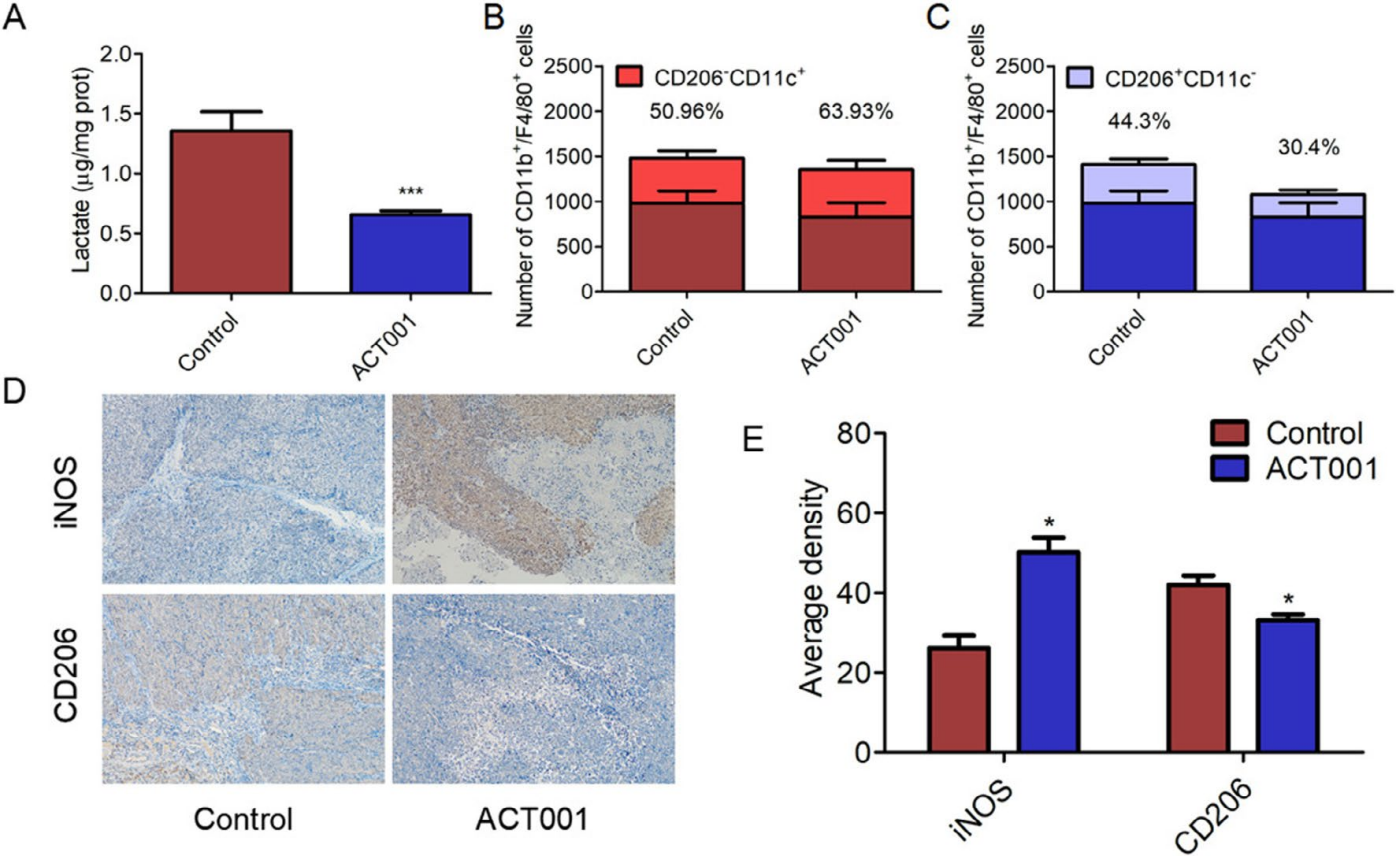
ACT001 reduced lactate levels, suppressed M2 macrophage polarization, enhanced M1 macrophage proportion, and stimulated anti‐tumor immunity in vivo. (A) Lactate concentration in tumors from control and ACT001‐treated groups. (B) Stacked bar chart depicting the number of infiltrated CD11b^+^/F4/80^+^ and CD11b^+^/F4/80^+^/CD206^−^/CD11c^+^ cells in tumors from control and ACT001 groups. (C) Stacked bar chart showing the number of infiltrated CD11b^+^/F4/80^+^ and CD11b^+^/F4/80^+^/CD206^+^/CD11c^−^ cells in tumors from control and ACT001‐treated groups. (D) IHC images of iNOS and CD206 in tumors from control and ACT001‐treated groups. (E) Quantitative analysis of iNOS and CD206 staining in tumor tissues. (*n* ≥ 3, *p < 0.05, **p < 0.01, ***p < 0.001).

Lactate produced by tumor cells contributes to the immune‐cold TME and converts recruited macrophages into M2‐polarized tumor‐associated macrophages. This study investigated the effects of ACT001 on lactate levels and macrophage infiltration in SCLC tumors. The results indicated that ACT001 significantly reduced lactate content in tumor tissue compared to the control group (Figure 8A). Flow cytometry analysis revealed that F4/80^+^CD11b^+^ macrophages were more prevalent in SCLC tumors than in ACT001‐treated tumors. ACT001 effectively decreased the proportion of the M2 phenotype (F4/80^+^/CD11b^+^/CD206^+^/CD11c^−^) while increasing the proportion of the M1 phenotype (F4/80^+^/CD11b^+^/CD206^−^/CD11c^+^), potentially owing to reduced intratumoral lactate levels (Figure 8B,C).

M1 macrophages promote anti‐tumor activity by converting arginine into nitric oxide through inducible nitric oxide synthase (iNOS). Conversely, M2 macrophages inhibit inflammatory responses by expressing CD206 molecules, facilitating tumor progression. IHC experiments were conducted to determine whether ACT001‐induced inhibition of lactate formation could induce an anti‐tumor immune response in vivo. IHC images demonstrated increased inducible nitric oxide synthase expression in ACT001‐treated groups (Figure 8D,E), while CD206 expression was significantly decreased (Figure 8D,E). These findings suggest that ACT001 improves the immune‐cold TME by promoting M1 macrophage polarization and inhibiting M2 macrophage proliferation.

## 4 Discussion

Cancer cells reprogram their metabolism to enhance malignant progression and suppress anti‐tumor immunity [52]. This metabolic reprogramming primarily relies on glycolysis, leading to the accumulation of onco‐metabolites such as lactate, creating an acidic TME. In addition, the high energy demands of cancer cell growth result in a low‐oxygen, low‐energy, and low‐pH TME, which significantly affects the human immune system. For instance, lactate accumulation in the TME depletes CD8^+^ T cells [53], affects natural killer cell and macrophage functions [26, 27, 28, 29, 30, 31], and promotes tumor cell immune escape, further accelerating tumor progression. Previous research has suggested that inhibiting glycolytic enzyme expression in cancer cells can improve immune cell function within tumors by restoring normal glucose levels in the TME [54].

Consequently, targeting specific metabolic pathways has emerged as a promising strategy for activating anti‐tumor immune responses and exerting anti‐tumor effects. PGK1 is a key enzyme in glucose metabolism and is involved in various biological activities, including chemotherapy resistance, angiogenesis, epithelial–mesenchymal transition, autophagy initiation, mitochondrial metabolism, and other processes associated with tumor development. However, its function in SCLC has not been previously reported. Our study demonstrates for the first time that PGK1 promotes SCLC progression and that knocking down PGK1 can significantly inhibit lactate production, metastasis, and invasion of SCLC cells. This study elucidates a novel mechanism by which ACT001 regulates tumor metabolism and enhances anti‐tumor immunity. Specifically, ACT001 inhibits the production of lactate, a glycolytic metabolite, by targeting PGK1 and affecting its downstream p‐PGK1/p‐PDHK axis in SCLC cells. ACT001 treatment reduces lactate production within tumors, decreases M2 macrophage infiltration, and enhances the function of M1 macrophages. Our research demonstrates that ACT001 improves the immune microenvironment of SCLC by promoting M1 macrophage polarization, supporting further investigation into combining ACT001 with ICIs to combat ICI resistance in SCLC. Additionally, ACT001 delays SCLC growth and metastasis both in vivo and in vitro, indicating its dual capacity to enhance the TIME and directly target tumor cells. These findings suggest that ACT001 is a promising drug candidate for SCLC treatment.

In conclusion, this study reveals that the natural product derivative ACT001 exhibits potential cytotoxicity against SCLC cells and regulates tumor glycolysis and anti‐tumor immunity by targeting PGK1. These findings provide a strong pharmacological rationale for using ACT001 as a monotherapy or in combination with ICIs for the treatment of SCLC.

## Author Contributions

**Xiao‐Jing Ding:** investigation, methodology, writing – original draft. **Ting Mei:** methodology. **Xiao‐Nan Xi:** methodology. **Jing‐Ya Wang:** methodology. **Wen‐Jing Wang:** investigation. **Yue Chen:** writing – review and editing, funding acquisition. **Ya‐Xin Lu:** methodology, conceptualization, funding acquisition. **Ting‐Ting Qin:** methodology, conceptualization, funding acquisition. **Ding‐Zhi Huang:** methodology, conceptualization, funding acquisition.

## Conflicts of Interest

The authors declare no conflicts of interest.

## Supporting information


Data S1.

## Data Availability

The data that support the findings of this study are available on request from the corresponding author.
