# Supplementary material for: ACT001 Suppresses the Malignant Progression of Small‐Cell Lung Cancer by Inhibiting Lactate Production and Promoting Anti‐Tumor Immunity
Source: Thorac Cancer. 2025 Feb 27;16(5):e70028. doi: 10.1111/1759-7714.70028 (PMC11868026; doi:10.1111/1759-7714.70028)
Supplement: Supplementary file 1 — Data S1. [file TCA-16-e70028-s001.docx]

**ACT001 suppresses the malignant progression of small-cell lung cancer by inhibiting lactate production and promoting anti-tumor immunity**

Xiao-Jing Ding ^1^, Ting Mei ^2, 3, 4, 5^, Xiao-Nan Xi ^1, 6^, Jing-Ya Wang ^2, 3, 4, 5^, Wen-Jing Wang ^1^, Yue Chen ^6, 7^, Ya-Xin Lu ^6, 7,^ **^*^**, Ting-Ting Qin ^2, 3, 4, 5,^ **^*^**, Ding-Zhi Huang ^2, 3, 4,^ ^5,^ **^*^**

^1^ College of Pharmacy, Nankai University, Tianjin 300350, China;

^2^ Tianjin Medical University Cancer Institute & Hospital, National Clinical Research Center for Cancer, Tianjin 300060, China;

^3^ Key Laboratory of Cancer Prevention and Therapy, Tianjin 300060, China;

^4^ Tianjin’s Clinical Research Center for Cancer, Tianjin 300060, China;

^5^ Department of Thoracic Oncology, Tianjin Lung Cancer Center, Tianjin Cancer Institute & Hospital, Tianjin Medical University, Tianjin 300060, China;

^6^ State Key Laboratory of Medicinal Chemical Biology, Nankai University, Tianjin 300350, China;

^7^ College of Chemistry, Nankai University, Tianjin 300350, China.

*****Correspondence to:

Ding-Zhi Huang & Ting-Ting Qin, Tianjin Medical University Cancer Institute & Hospital, National Clinical Research Center for Cancer, Huanhu West Road, Tianta Street, Hexi District, Tianjin, 300350, China. Email: [huangdingzhi@tjmuch.com](mailto:(dingzhih72@163.com);) & [qintingting@tjmuch.com](mailto:qintingting@tjmuch.com);

Ya-Xin Lu, State Key Laboratory of Medicinal Chemical Biology, Nankai University, No. 38 Tongyan Road, Jinnan District, Tianjin 300350, China. Email: [yaxinlu@nankai.edu.cn](mailto:yaxinlu@nankai.edu.cn).**METHODS**

**Cell lines**

NCI-H1688 and NCI-H446 cell lines were kindly provided by Accendatech Co., Ltd (Tianjin, China). GFP-H1688 and THP-1 were purchased from ATCC. All cell lines were kept in RPMI-1640 (C11875500BT, Gibco) containing 10% fetal bovine serum (FBS) and cultured in a 5% CO_2_ incubator at 37 °C.

**MTT assay**

SCLC cells were seeded in a 9-well plate at a density of 2000 cells per well and then treated with graded amount of ACT001 (Accendatech Co., Ltd, Tianjin, China) and MCL (Accendatech Co., Ltd, Tianjin, China). After 72 h, 10 µL 5 mg/ml MTT (298-93-1, Solarbio, China) solution was added to each well and incubated at 37 °C for an additional 4 h. The supernatant was completely removed from each well. Then, 100 µl of DMSO was added to dissolve the formazan crystals. The absorbance was measured at 490 nm by a microplate reader (MD, Austria).

**Clone formation experiment**

SCLC cells with a density of 200 cells per well were seeded in a 6-well plate. Cells exposed to different doses of ACT001 for 24 h and then incubated for 10 days. Then, the cells were washed with PBS, fixed with 4% paraformaldehyde and subsequently colored with crystal violet solution. After the residual staining solution was washed away, a microscope was used to capture the number of cell clones formed.

**EdU cell proliferation assay**

1 × 10^5^ cells/well of cells were seeded into confocal dishes. After the cells were incubated with ACT001 at indicated concentration for 24 h, EdU assay was conducted by instructions of BeyoClickTM EdU-555 cell proliferation detection kit (C0075L, Beyotime). The LSM800 laser confocal microscope (Zeiss, Germany) was used to take the images.

**Wound healing assay**

5×10^5^ cells/well of SCLC cells were seeded in 12-well plates and grown to attach at cell incubator. Then these cells were wounded with 200 μL plastic pipette tips and washed 3 times with PBS. Cells were treated with ACT001 at indicated condition for 48 h and the closure of wound was measured by microscope.

**Transwell cell invasion experiment**

After 24 h incubation with ACT001, SCLC cells were seeded into a matrigel-coated transwell cells. 24 h later, each well was washed twice by PBS. Then the cells were fixed in 4% paraformaldehyde and stained with 1% crystal violet (548-62-9, Solarbio, China). Then, the cells were captured by a microscope and counted in three independent fields.

**Western blot assay**

SCLC cells were seeded in 6-well plates and incubated until 90% confluent. The cells were exposed to ACT001 at indicated concentration for 24–48 h. After that, the cells were harvested. 100 μL RIPA buffer was added to lyse cell. The supernatant was collected and used as protein extracts. After protein quantification, 20 µg protein were denatured and separated by SDS-PAGE. Then, the gels were transferred to PVDF membranes. After blocking with 2.5% non-fat milk in tris-buffered saline-tween (TBST) solution for 2 h, the membranes were incubated with different primary antibodies at 4 °C for 12 h. After washing, the bands were incubated with secondary antibodies at room temperature for 1 h. Finally, the bands were detected with the ECL system (Millipore, Billerica, USA).

**Lactate formation and glucose uptake assay**

The l-lactate assay kit (AB65330, Abcam, Japan) and glucose assay kit (GAGO20, Sigma-Aldrich, Darmstadt, Germany) were used to detect lactate production levels and the glucose uptake in SCLC cells. The experiment was carried out according to the protocol provided by the kit.

**In vitro pull-down assay**

NCI-H1688 or NCI-H446 cells were harvested and lysed in PBS. The lysis solution was collected followed by addition of 20 µM biotin-labeled MCL probe (Biotin-MCL) (Accendatech Co., Ltd, Tianjin, China). The mixture was pre-incubated overnight at 4 ℃. After that excessive pre-cooled methanol was added and the mixture was incubated at −20 ℃ for 30 min to precipitate the protein. The precipitated proteins were re-dissolved and incubated with streptavidin beads (20228, Thermo scientific, USA) for another 4 h at 4 ℃. After that the streptavidin beads were washed six times with 0.1% SDS PBS buffer, the bead-bound proteins were eluted, separated by SDS-PAGE, and visualized by silver staining, proteomic analysis or western blot assay.

**Cellular thermal shift assay (CETSA)**

The SCLC cells were collected and lysed. The cell extract was divided into two groups, one group of cell lysate was added to PBS as the control group, and the other group of cell lysate was added to 100 µM of MCL. Then, the cell lysates were incubated at room temperature for 2 h. After incubation, the two groups of cell lysates were divided into three parts and transferred to a new PCR tube, respectively. MCL treatment group and a control group were heated in parallel using a PCR machine for 3 min at 49, 53, and 59 ℃, respectively. Then, the supernatant was collected after the sample was centrifuged. Western blot assay was used to test the ability of MCL to stabilize target proteins.

**PGK1 enzyme activity detection**

2 ng recombinant wild-type PGK1 protein was incubated with or without the different concentration of MCL at room temperature for 30 min. After incubation, 100 µl reaction system including 4 mM 3-PG (P8877, Sigma-Aldrich, USA), 3 mM ATP (tlrl-atp, Invivogen, France), 400 µM NADH (N8129, Sigma-Aldrich, USA) and 200 nM GAPDH (P02436, Solarbio, China) was added to PGK1 protein with or without MCL in a 96-well plate. The change of NADH level was read at 340 nm in kinetic mode for 30 min. PGK1 activity was measured as the rate of consumption of NADH. Inhibition rate (%) = (OD340 nm control-OD340 nm MCL) / OD340 nm control × 100%.

**Determination of binding site of MCL on PGK1**

200 µg MCL and 20 µg recombinant PGK1 protein were incubated at 4 °C overnight. The reaction system was separated using SDS-PAGE. The PGK1 band in gel was cut and digested with trypsin. The nano-LC-MS/MS experiments were performed using Triple-quad Ion-trap and Orbitrap fusion (Thermo Fisher Scientific, USA). Mass spectrometric data were analyzed using Proteome Discoverer software with the SEQUEST search engine (Thermo Scientific).

**Immunofluorescence assay**

Co-location assay: SCLC cells were implanted in confocal dishes with 10% confluence. After 12 h, the cells were washed 3 times with PBS and fixed with 4% paraformaldehyde for 30 min. Then the cells were added to MCL probes and incubated overnight. Triton X-100 was added to the cells to penetrate the cell membrane. Then the cells were blocked with 5% fetal bovine serum in PBS for 2 h. After that, cells were incubated with anti-PGK1 antibody at 4 °C overnight. Then, FITC coupled goat anti-rabbit IgG was incubated in the dark at RT for 1 h. Next, the cells were treated with DAPI for 5 min. The samples were pictured by a laser scanning microscope (TCS SP8, Leica Microsystems CMS).

Mitochondrial translocation: SCLC cells were pre-treated with or without ACT001 before being treated with hypoxia for 6 h. Then the immunofluorescence assay was performed using an anti-PGK1 antibody, MitoTracker (C1049B, Beyotime, China) and DAPI (P0131, Beyotime, China). The samples were pictured by a laser scanning microscope (TCS SP8, Leica Microsystems CMS).

**PGK1 knockdown experiment**

PGK1 siRNA was used to knock down the expression of PGK1 in SCLC cells. PGK1 siRNA and the negative control siRNA were purchased from Tsingke Biotechnology Co., Ltd (China). Transfection was carried out using Lipofectamine 2000 reagent (Invitrogen, Carlsbad, USA) in accordance with the manufacturer’s protocol. The knockdown effect was validated using western blot experiments.

**Tumor xenograft model**

1. week-old female nude Balb/C mice were raised in an SPF environment for 7 days. When the weight of mice reached 18–22 g, NCI-H1688 cells were dispersed at a density of 1×10^8^ cells/ml in PBS containing 20% Matrigel (BD, USA). 100 µl cell suspension was implanted in the right armpit of mice. Tumor volume (V) is measured and calculated as follows: V = (ab^2^)/2, where “a” represents the long diameter of the tumour, and “b” represents the short diameter of the tumour mass. When the tumor volume reaches 40–70 mm3, the mice are randomly divided into PBS group and ACT001 administration group. The ACT001 group was orally administered once a day at a dose of 200 mg/kg. The body weight and tumor volume of mice are measured every two days, and the first administration is recorded as the first day. On the 21st day, the mice were euthanized by cervical dislocation and then tumor tissues were removed.

**Immunohistochemical analysis (IHC)**

The tumors were fixed with formalin, embedded in paraffin, and cut into 5-µm sections for IHC. The sections were stained with anti-PDHK1(Phospho-Thr338) rabbit polyclonal antibody, anti-CD206 antibody (18704-1-AP, Proteintech, China), and anti-iNOS antibody (22226-1-AP, Proteintech, China).

**Intratumoral macrophage analysis**

Tumor tissue was minced in DMEM and digested in Hanks containing 0.5 mg/ml collagenase I (BS163, Biosharp, China), 0.1 mg/ml dispase (S25046, Shanghaiyuanye Bio-Technology Co.,Ltd, China) and 0.1 mg/ml DNase I (BS137, Biosharp, China). After 1 h of digestion at 37 °C, the tumor tissue was filtered into 15 ml centrifuge tube using a 70 µm membrane (BS-70-XBS, Biosharp, China). Then the cells were collected in 30% Percoll. The cells were mixed and centrifuged at 14000 g for 5 min. After that, the cells were collected and added to the red blood cell lysate. PBS containing 2% FBS was added to terminate lysis. Finally, the samples were stained by APC anti-mouse F4/80 Antibody (123115, Biolegend, USA), PE anti-mouse/human CD11b Antibody (101207, Biolegend, USA), PerCP anti-mouse CD11c Antibody (117325, Biolegend, USA), and FITC anti-mouse CD206 (MMR) Antibody (141703, Biolegend, USA) on ice for 1 h in the dark. After staining, flow analysis was performed using BD flow cytometry. Data were analyzed using FlowJo software.

**Intratumoral lactate analysis**

The experiment was carried out according to the protocol provided by the l-lactate assay kit (AB65330, Abcam, Japan).

**Conditioned medium (CM) preparation and macrophage polarization**

NCI-H1688 cells treated with or without ACT001 were incubated in serum-free medium for 48 h and then centrifuged at 10,000 rpm for 5 min, after which supernatants were collected as conditioned medium and stored at − 80 ℃. The THP-1 cells were differentiated into M0 macrophages by incubating in 320 nmol/L phorbol myristate acetate (PMA, Solarbio, China) for 18 h. To generate M2-polarized macrophages, THP-1 cells were treated with 320 nmol/L PMA for 12 h and then cultured with 100 nmol/L PMA plus 20 ng/mL IL-4 and IL-13 (Peprotech, NJ, USA) for a further 48 h. Then the markers CD206 and arginase 1 (Arg1) of M2 macrophages were detected. The anti-CD206 primary antibody (60143-1-Ig, Proteintech, China), the anti-Arg1 primary antibody (16001-1-AP, Proteintech, China).

**In vivo metastasis model and drug treatment**

6-week-old female nude Balb/C mice were raised in an SPF environment for 7 days. GFP-H1688 cells were dispersed at a density of 1×10^7^ cells/ml in PBS. 200 µl cell suspension was injected into the tail veins of mice. 12 mice with the same method of construction of metastasis models were randomly divided into 2 groups (6 in each group). The ACT001 group was orally administered once a day at a dose of 200 mg/kg. The control group was treated with an equal amount of PBS. Mice were sacrificed and dissected 3 weeks after injection to observe metastatic nodules in possible target organs by using an IVIS Spectrum instrument (PerkinElmer, USA).

**Statistical analysis**

The data were presented as mean ± standard deviation (SD). Statistical analysis was calculated by the unpaired two-tailed Student’s t-test. Statistically significant results were those with a P < 0.05.
